# Fuzzy logic: A “simple” solution for complexities in neurosciences?

**DOI:** 10.4103/2152-7806.77177

**Published:** 2011-02-26

**Authors:** Saniya Siraj Godil, Muhammad Shahzad Shamim, Syed Ather Enam, Uvais Qidwai

**Affiliations:** Faculty of Health Sciences, Medical College, Aga Khan University, Karachi, Pakistan; 1 Section of Neurosurgery, Department of Surgery, Aga Khan University Hospital, Karachi, Pakistan; 2 Department of Computer Science and Engineering, Qatar University, Qatar

**Keywords:** Fuzzy logic, neurosciences, neurology, neurosurgery, psychiatry

## Abstract

**Background:**

Fuzzy logic is a multi-valued logic which is similar to human thinking and interpretation. It has the potential of combining human heuristics into computer-assisted decision making, which is applicable to individual patients as it takes into account all the factors and complexities of individuals. Fuzzy logic has been applied in all disciplines of medicine in some form and recently its applicability in neurosciences has also gained momentum.

**Methods:**

This review focuses on the use of this concept in various branches of neurosciences including basic neuroscience, neurology, neurosurgery, psychiatry and psychology.

**Results:**

The applicability of fuzzy logic is not limited to research related to neuroanatomy, imaging nerve fibers and understanding neurophysiology, but it is also a sensitive and specific tool for interpretation of EEGs, EMGs and MRIs and an effective controller device in intensive care units. It has been used for risk stratification of stroke, diagnosis of different psychiatric illnesses and even planning neurosurgical procedures.

**Conclusions:**

In the future, fuzzy logic has the potential of becoming the basis of all clinical decision making and our understanding of neurosciences.

## INTRODUCTION

Man is God’s most complex creation. Clinical judgment for the diagnosis and management of mans’ diseases is an art. It can neither be acquired from textbooks alone, nor can it be taught, but has to be developed slowly through years of observation and experience. This is because unlike other professions, which thrive on calculations based on yes/no or present/absent, very little is clearly black and white in clinical medicine. Most clinical scenarios present in shades of gray. Instead of “present or absent”, patients’ symptoms are described using terms like “never, rarely, sometimes, often, most of the times, always, etc”. Moreover, each specific symptom may also be graded as “mild, moderate or severe”. This is compounded by the fact that most symptoms are experienced and described differently by patients and many symptoms may overlap in the same patient. Each individual patient may also have a multitude of characteristics other than the disease, rendering it unique in itself. Medical problems, therefore, cannot be generalized and analyzed using Aristotelian or binary logic, and an analytical program is desperately required which could integrate this complex network of problems and devise individualized solutions. Fuzzy logic is the nearest response to the call. It has the potential of combining human heuristics into computer-assisted decision making. Imagine combining the experience of five university professors with all the current literature and developing a software that can calculate probabilities based on this, tailored specifically for each individual patient. Fuzzy logic can do all that.

The concept was first introduced by Lotfi Zadeh in 1965.[111] He defined fuzzy logic as “a class of objects with a continuum of grades of membership”.[111] It accounts for all the complexities and variations in patients and results in a statistical analysis which is appropriate for an “individual”, unlike evidence-based medicine, which is applicable to a group of patients.[44] It enables the scientific community to look into all shades of gray and determine the grade and severity of the disease. Fuzzy logic is a well-established concept in mathematics and engineering but its usefulness in medicine was not realized till the last decade. A recent review highlighted that the medical publications on fuzzy logic increased from 2 per year in 1991 to 175 per year in 2002.[99] Till last year, a Medline search using the keyword “fuzzy logic” generated around 1600 publications,[95] but a recent Medline search generated a total of 2448 articles, out of which more than 300 were published during last year. This reflects that the use and applicability of fuzzy logic is accelerating at a significant pace in medical and scientific community.

## WHAT IS FUZZY LOGIC?

Fuzzy logic is a multi-valued logic which was introduced by Zadeh in order to deal with vague and indecisive ideas.[111] It has been described as an extension to the conventional Aristotelian and Boolean logic as it deals with “degrees of truth” rather than absolute values of “0 and 1” or “true/false”. Fuzzy logic is not like a computer software which understands only binary functions or concrete values like 1.5, 2.8, etc; instead, it is similar to human thinking and interpretation and gives meaning to expressions like “often”, “smaller” and “higher”. Fuzzy logic takes into account that real world is complex and there are uncertainties; everything cannot have absolute values and follow a linear function.

### Characteristics of Fuzzy Logic

There are a few basic principles of fuzzy logic which were laid down by Zadeh in 1992:[110]

- Exact reasoning is viewed as a limiting case of approximate reasoning.
- Everything is a matter of degree.
- Knowledge is interpreted as a collection of elastic, fuzzy constraints on a collection of variables.
- Inference is viewed as a process of propagation of elastic constraints.
- Any logical system can be “fuzzified”.

### Fuzzy Sets

**Figure 1:**
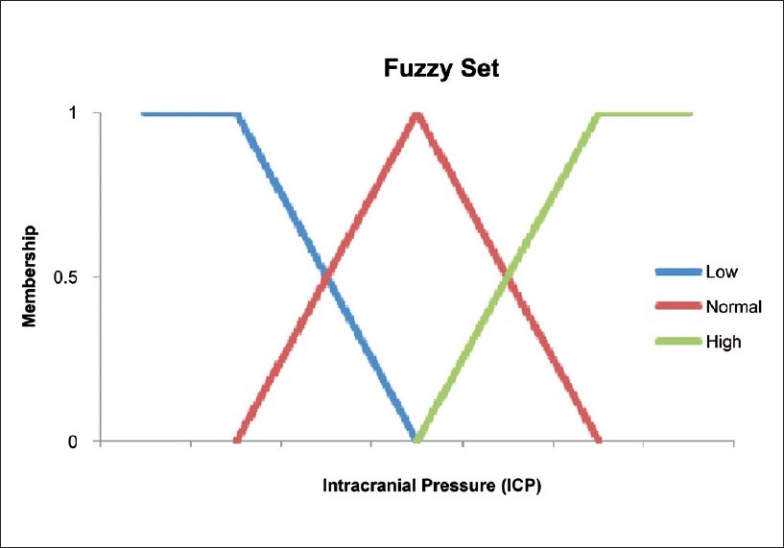
Fuzzy sets: low, medium and high

A classical set of binary logic has “crisp” boundaries whereas fuzzy sets have fuzzy or imprecise boundaries. A fuzzy set consists of linguistic variables where values are words and not numerical.[110–111] For example, intracranial pressure (ICP) can be defined as low, normal or high. Thus, ICP is a linguistic variable where the values have fuzzy margins and can overlap each other [Figure 1]. The transition from one value to another is gradual and each value is given a membership function which represents the degree to which it belongs to that value. A fuzzy set can be represented by the following equation:[5]

$$A\;=\;\left\{\left(\mathrm{\{(x},\;\mu_{A}\left(\mathrm{(x))}\right)\right)\;|\;x\;\in \;\mathrm{X\}}\right\}$$

where A is a fuzzy set in X and μ_A_(x) is the membership function, which can have any value between 0 and 1 inclusive.

Membership functions overlap each other as evident in Figure 1. Thus, a value for ICP can be both low and normal to a certain degree. Membership functions are not equivalent to probabilities. A membership value of low ICP does not signify that there is a certain probability of having low ICP or not; instead it is the degree to which it is a low ICP.

### Fuzzy Rules

Fuzzy rule is based on “if…then” rule and connects the different input and output fuzzy variables.[110] It can be expressed as:

if is x *A* then y is *B*

where *A* is the antecedent and *B* is the consequent. Fuzzy rules are similar to common sense rules as they resemble human thinking and are based on human experience. For example, in order to control ICP in a patient with traumatic brain injury, sedation is often required but needs to be carefully monitored. A simple rule can be, “If the ICP is high, increase propofol infusion”, or “If the ICP is low, stop propofol infusion”. These rules are based on collective experience of specialists in the field as well as available literature. Thus, as more fuzzy rules and sets are obtained from various sources, uncertainties are potentially reduced.

### Fuzzy Reasoning

Fuzzy reasoning is also called approximate reasoning and is the process of drawing conclusions from fuzzy sets and fuzzy rules.

### Fuzzy Inference System

**Figure 2:**
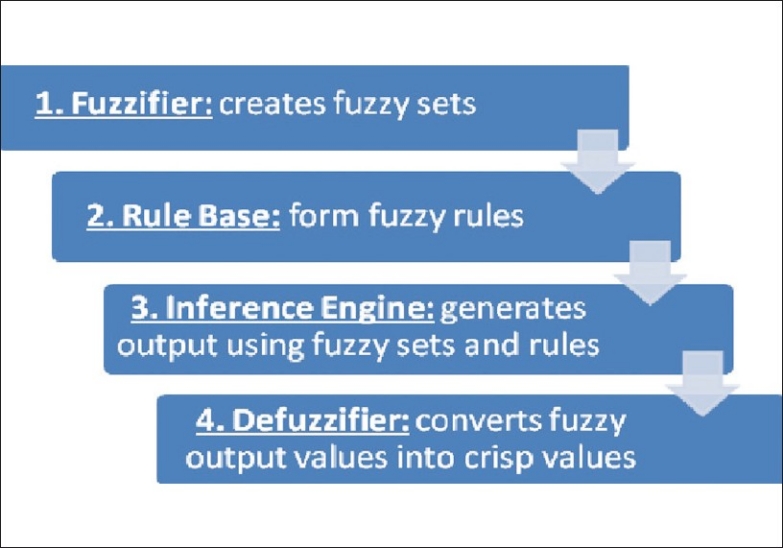
Components of fuzzy inference system

Fuzzy inference system (FIS) is a framework which is based on fuzzy sets, fuzzy rules and fuzzy reasoning.[78] It has four main components including fuzzifier, rule base, inference engine and defuzzifier[106] [Figure 2]. The fuzzifier creates fuzzy sets from “crisp” values like a fuzzy set for ICP will be divided into “low, normal and high” and a fuzzy set for propofol infusion will be divided into “stop, decrease and increase”. Next, the fuzzy rules are formed based on these two input fuzzy sets: “If the ICP is low, stop propofol infusion”, “If the ICP is normal decrease propofol infusion” and “If the ICP is high, increase propofol infusion”. The inference engine applies all the fuzzy rules on the fuzzy sets to determine the resultant fuzzy output. If a “crisp” output value is required, the process of defuzzification converts the fuzzy output into a “crisp” output value by determining the center of mass of the combined, overlapping membership functions.

## EXAMPLES OF A FUZZY LOGIC NETWORK

Microdiskectomy is a common surgical procedure performed for low back pain and radiculopathy due to disc herniation. It provides symptom relief in most patients. However, a few patients fail to improve after this surgical procedure and fuzzy logic based FIS was used by Shamim *et al*, to predict this group of patients with failed microdiskectomy.[95]

A retrospective review of 501 patients who underwent microdiskectomy was done. A total of 16 variables from a list of 54 variables were classified as risk factors for failed microdiskectomy by an expert in the field. These variables were taken as membership function and the degrees of membership were defined. A rule base of 11 fuzzy rules was formed and each rule formed a decision bar which together made up the total decision surface. The centroid of each decision surface formed the basis of FIS decision. The output variable defined the risk of failed microdiskectomy as “very low”, “low” or “high” risk. The sensitivity and specificity of the FIS was calculated by comparing these results of FIS with the actual outcome of all patients at a six-month postoperative follow-up. The sensitivity and specificity of this FIS was found to be 88% and 86%, respectively.[95]

Another example highlights the application of FIS in predicting trauma-related mortality.[109] Retrospective data of 150 trauma patients was collected including GCS, systolic blood pressure and different trauma scores (Injury Severity Score [ISS], Revised Trauma Score [RTS], A Severity Characterization of Trauma [ASCOT] and Trauma and Injury Severity Score [TRISS]) at arrival, one hour after resuscitation and at the time of ICU admission. By using the different trauma scores, mortality prediction was calculated at the time of arrival, 1 hour after resuscitation and at the time of ICU admission. For fuzzy logic, the membership functions of GCS and systolic blood pressure (e.g. very low, low, normal, high) as well as change in GCS and systolic blood pressure at 1 hour and at the time of ICU admission (e.g. decrease, stationary, increase) were defined. Different fuzzy rule blocks were made using these membership functions. For example very low GCS and systolic blood pressure at arrival and a decrease in GCS and blood pressure after resuscitation predict very high mortality. The FIS designed integrated all these membership functions and fuzzy rules to predict mortality of these patients using Mamdani center-of-gravity algorithm. FIS (ROC: 0.9247) performed better than other conventional scoring systems (ROC: 0.9033).

One of the most important uses of fuzzy logic is in drug delivery devices. Special fuzzy logic controllers have been designed for use in anesthesia and intensive care units. One of the studies used auditory evoked potential as a measure of depth of anesthesia and the fuzzy controller administered a certain amount of drug based on it.[8] Similarly neuromuscular blocking agents are administered during surgery by monitoring the response at ulnar nerve; if the response at ulnar nerve is greater, more neuromuscular blocker is administered by the fuzzy controller.[85]

All these examples clearly indicate that fuzzy logic networks and systems can easily solve various complex clinical problems.

## ADVANTAGES AND DISADVANTAGES OF FUZZY LOGIC

Fuzzy logic is a solution to complex problems in all fields of life, including medicine, as it resembles human reasoning and decision making. It looks into all shades of gray and answers uncertainties and ambiguities created by human language where everything cannot be described in precise and discrete terms. Fuzzy systems help define disease extent and severity and answer questions related to individual patients taking into account their risk factors and co-morbidities.

On the other hand, it has a number of disadvantages too. It is tedious to develop fuzzy rules and membership functions and fuzzy outputs can be interpreted in a number of ways making analysis difficult. In addition, it requires lot of data and expertise to develop a fuzzy system. It does not give generalizable results and the program has to be run for each individual patient. Therefore, its clinical applicability and utilization is difficult without the availability of preprogrammed softwares for different pathologies and the basic training of clinicians to use these programs.

## FUZZY LOGIC IN MEDICINE

The application on fuzzy logic in medicine gained momentum in last two decades[76,99] when the usefulness of this technique was realized to correlate with the fuzzy nature of this field. In this era of technological advancements, most of the human workforce has been replaced by machines and robots and fuzzy logic is the means by which a system can be formulated, whereby machines can perform tasks by using rules similar to human reasoning and logic.

All the disciplines of medicine have used fuzzy logic in some way. The various applications include: assessing the effectiveness of drugs,[75] early detection of diabetic retinopathy and neuropathy,[112] treatment of tropical diseases[73] and diagnosis of diabetes, cardiac, renal and liver diseases[3,51,54] where it has shown an accuracy of 79.37% in diagnosing diabetes and 97.55% in diagnosing dermatological diseases.[52] In pulmonary medicine it has been used for chronic obstructive pulmonary disease, evaluating pulmonary function tests, as well as for ventilator support of patients in intensive care unit (ICU).[13,30,70,102] In ICU setups, FIS has been shown to monitor patients, control blood pressure, provide adequate analgesia and anesthesia, assess ventilation requirements and even control ventilator settings, etc.[14,25,29] FIS has also been shown to help in controlling tidal volume, maintaining end-tidal PCO_2_ and controlling administration of neuromuscular blockade during surgery,[28,60–61,71–72,90] as well as maintenance of depth of anesthesia and optimum conditions for surgery.[40] In oncology, detection of cancers, including lung, breast and prostate cancer, can be aided with fuzzy logic.[64,93–94] It can also predict surgical outcomes and prognosis in malignancies[46,92] and can be used to decide radiotherapy margins.[67] It is also helpful in analyzing PET scan images for quantification of cancers.[16]

Fuzzy logic is not only applicable to clinical medicine, but it has been a useful statistical tool in basic sciences and bioinformatics as well.[99] DNA sequencing, studying the complete genome and differences between polynucleotides and understanding various signaling pathways and cell signaling networks is possible by using fuzzy logic.[6,24,38,66,82,100]

## FUZZY LOGIC IN NEUROSCIENCES

In comparison to applicability of fuzzy logic in medicine and basic sciences, the concept is still new in the field of neurosciences. This was clearly highlighted in the review published on fuzzy logic where the contribution to the literature on fuzzy logic was much less from neurosciences as compared to other disciplines of medicine.[59] However, the last decade has witnessed the acknowledgment of usefulness of fuzzy logic in various branches of neurosciences including basic neurosciences, neurology, neurosurgery, neuroradiology, psychiatry and psychology.[15,95,99]

### Basic Neurosciences

Fuzzy model has been shown to be an effective tool for research related to neuroanatomy and has been used for imaging nerve fibers.[12,108] Controller systems based on fuzzy logic are also capable of controlling electrical responses, like potential difference and current, of nerve fibers.[4] Additionally, it has also been used in tracking eye movements and characterizing horizontal and vertical nystagmus[9] and studying neural circuits in primary visual cortex.[36] It can be utilized in understanding the sensorimotor behavior of individuals as well as other complex neurophysiological concepts and neuron circuitry.[80]

### Neurology

Fuzzy logic has been used in the diagnosis, management and outcome prediction of common neurological diseases with considerable success. Stroke is a multifactorial disease and the causal relationship is complex. Jobe *et al*, showed that all probabilistic-statistical methods are ineffective in explaining this complex relationship except fuzzy model, which is a good tool to understand the disease causality.[43] Another study on stroke patients, comparing statistical versus fuzzy measures, also concluded that fuzzy measures are a better representation of the actual, individual patient as compared to other statistical measures which apply to a group of patients and not individuals.[35] Fuzzy logic has been shown to be superior to evidence-based medicine in complex clinical scenarios[32–33] and cases where individual factors of patients are important to consider, for example, administration of warfarin in stroke patients.[31,34] Fuzzy clustering method has also been applied on stroke patients to analyze different biomechanical forces and devise an effective rehabilitation program according to their specific requirements.[2]

FIS has also been implicated in the study and interpretation of EEG. Aarabi *et al*, developed a FIS, which was highly sensitive (sensitivity: 98.7%) in detecting seizures via intracranial EEG.[1] It is a remarkable finding as the detection of seizures by FIS was comparable to the detection by experts. Fuzzy index can distinguish between EEG signals from normal individuals and those from epileptic patients[101] and help in the diagnosis of epilepsy with a sensitivity of 84.9%.[18,22] EEG analysis using FIS can also be used to determine the depth of anesthesia.[53] Fuzzy system has also been shown to accurately identify different stages of sleep 84.6% of the times based on EEG findings.[42]

A fuzzy logic based biofeedback system through EMG has been found to significantly change the activation pattern of trapezius muscle during active and passive shoulder movements (*P* value <0.05).[88] The feedback significantly changes the spatiotemporal activity of the trapezius muscle (*P* value <0.05).[87] In a study by Kocer *et al*, EMG signals from various patients were classified and analyzed with the help of neuro-fuzzy system. This system can help in the diagnosis of various neuromuscular diseases, including myopathies and neuropathies, based on EMG findings.[47] A technique introduced by Chauvet can be used to study the physiological properties of muscles without using invasive methods.[19] By this technique, the EMG signals, classified using fuzzy logic, were decomposed into motor unit action potential trains and the error rate was found to be only 1.37% as 21 action potential trains were detected using this technique as compared to 29 detected by neurophysiologists.[19]

Fuzzy logic and application of different fuzzy rules can successfully predict and model human stance and gait which is controlled by the length of limb, its orientation and trunk attitude.[39] Zhang *et al*, have also developed a motor system for functional electric stimulation locomotion which is controlled by neural network and fuzzy logic.[113] A simulation model consisted of 7 segments and 18 muscles and had a satisfactory performance. Thus, this system can be used for locomotion in paraplegic patients. Recently, programmed logic controllers have been developed which use EEG signals from motor cortex as control signals and can be fitted into devices like wheel chairs and electronic machines, which can be used by disabled people.[41]

### Neurosurgery

The concept of fuzzy logic has been applied in neurosurgical ICUs for precise control of different parameters like ICP and blood pressure. Fuzzy logic based controllers are found effective at maintaining stable ICP via varying propofol infusion rates.[37,83–84,97] Fuzzy logic can also be used to estimate ICP non-invasively by using different parameters like cerebral blood flow velocity and arterial blood pressure with the difference between the actual and estimated ICP being only 5.7 mmHg.[91]

A fuzzy Glasgow Coma Scale (GCS) has been introduced and was used in a study conducted on traumatic brain injury patients in India.[10] The study showed that the non-specificity of classical and fuzzy GCS is comparable, as fuzzy GCS was able to effectively predict full cognitive recovery. Eye, visual and motor stimuli were all found to be significantly associated with cognitive recovery.[10] For general trauma, Kilic *et al*, have devised a FIS which has been shown to predict trauma-related mortality (ROC: 0.925) as well as conventional systems (ROC: 0.903).[109] Outcome of surgeries can also be predicted with the help of fuzzy logic. It has been shown that FIS has a sensitivity of 88% and specificity of 86%, with positive and negative predictive value of 0.36 and 0.98, respectively, for predicting poor outcomes in patients undergoing lumbar disc surgery.[95] Fuzzy logic has been used to develop a successful software for evaluating neurosurgical patients with brain tumors in clinic and estimate the tumor volume which will determine the treatment plan.[56] The mean operating time for the software is just 16 minutes and operator variability is less than 1%.

Samejima *et al*, developed a screening tool for unruptured aneurysm using fuzzy logic, based on the data from a retrospective study and opinions of experienced neurosurgeons and this tool was shown to detect 12 new cases of unruptured intracranial aneurysm.[89] Recently, another tool has been developed to predict outcomes of patients with intracranial aneurysm.[57] This study showed that the fuzzy logic based predictive tool was effective as the predicted outcomes correlated with the actual outcomes. The NASA smart probe project has used fuzzy logic in the development of probes for real-time identification of gray and white matter in brain and differentiation between normal tissue and tumor cells.[11,86] Surgery for deep brain stimulation can also be planned by combining data from MRI images and expert opinions with the help of fuzzy logic.[105] This can result in targeting the exact anatomical area and increase the success of surgery. Hemm *et al*, showed that using fuzzy logic helps in better anatomical localization of structures and placement of electrodes for deep brain stimulation.[98]

Surgical planning for correcting spinal deformities is another area where fuzzy logic has been used effectively. An important decision for these surgeries is the level at which surgical correction is required. It is highly dependent on opinion of experienced surgeons, and using fuzzy logic, has now been integrated into a model to aid in surgical planning.[69] Miller *et al*, have designed a modular surgical instrument based on FIS which has been shown to reduce operating time by almost 7% by efficiently managing the workflow and handling of surgical instruments.[63] This instrument was designed for laparoscopic surgeries but can be applied to any form of surgeries and robotic systems.

Morphometric measurements and analyses of gray and white matter of spine can also be done using fuzzy logic in patients with spinal cord injury.[26] Ellingson *et al*, found that cervical spinal cord is significantly affected during injury to caudal spinal cord, diffusion is reduced in both gray and white matter but atrophy in white matter tracts, measured using FIS, is higher as compared to gray matter.[26]

## NEURORADIOLOGY

MRI is the most commonly used radiological investigation for diagnosis of brain tumors and stroke. A number of studies have been conducted to analyze MRI images using fuzzy models.[45,55,114] Fuzzy logic has a higher sensitivity of detecting small lesions of stroke and emboli and also identifying normal structures.[48,96] Fuzzy cluster can be used to accurately characterize gliomas into high grade and low grade (*P* value <0.001) and determine glioma volume before surgery.[27,65,77] The diagnosis of grade of gliomas, based on interpretation of MRI scans using fuzzy logic, has been shown to have an accuracy of 86.4%.[107] It is also able to detect brain tumor response to radiation therapy by measuring changes in volume after treatment.[103,104] Additionally, it can differentiate tumor tissue from surrounding edema and hemorrhage.[77,79] Apart from the tumor volume, fuzzy clustering can detect CSF and gray and white matter volume changes in children with hydrocephalus.[17]

Classification and segmentation of the cerebral hemispheres, cerebellum and deep structures of the brain can be performed by fuzzy systems using information from the anatomical atlas and MRI image characteristics.[7,20–21] Fuzzy connectivity has been used for segmentation and identification of black spots in multiple sclerosis.[23]

Till date, there are no specific diagnostic criteria for diagnosis of cortical malformation which is a common neurological problem leading to epilepsy, mental retardation and developmental delay in children. A recent paper has used fuzzy logic to incorporate expert opinions and formulate a system for diagnosis of cortical malformation.[5]

### Psychiatry and Psychology

A fuzzy logic based software has been designed to keep a track of psychiatric patients. It includes all the recent diagnostic criteria for different disorders and has complete details of the patients’ histories and follow ups which help in the management of these patients as well as assisting in research.[49] Diagnosis of sleep disorders is dependent on clinician’s experience. Fuzzy logic has been shown to aid in the recognition and diagnosis of sleep disorders by analyzing data of opinions from experts.[74]

Qing *et al*, developed a fuzzy logic based functional MRI model for the evaluation of depression and its severity.[81] The correlation value between this model and standard depression severity scale was 0.7886, which signifies that it can be used to evaluate the severity of depression as well as to track the course of illness. Fuzzy logic is a sensitive technique, with an error rate of just 5.93%, in assessing weight changes in schizophrenic patients on antipsychotic drugs.[50] It is also used in auditory electrophysiological monitoring of patients with schizophrenia.[^115^]

Fuzzy systems have been successfully developed to monitor drug response in patients with drug dependence. The example of one such model is use of citalopram in alcohol drug dependence where high correlation (*r* = 0.99, *P* value <0.001) was seen between the actual and predicted response rate.[68] Fuzzy logic has also been applied in study of psychology. It was used in the interpretation of whether perception of speech varies with using upright or inverted facial images or not.[62] It has also been used to develop a model of emotions where the influence of different events and experiences on emotional process can be studied.[58]

## FUTURE DIRECTIONS

The above discussion clearly highlights fuzzy logic as a sensitive and specific tool for various clinical problems. Although, a number of studies have been conducted on fuzzy logic, it is still largely underutilized in neurosciences. On one hand, where the concept has the potential of changing medical diagnosis and management completely, it remains to be seen how effectively it can be incorporated in routine clinical practice. If focused research is conducted, it is possible that in future neurophysiology labs will be reporting EMGs and EEGs with the help of fuzzy logic, ICUs will have fuzzy controllers for controlling blood pressure, ICP and ventilator settings, MRI scans will be analyzed by fuzzy logic softwares and neurosurgeries will be planned by FIS. However, change is always difficult to introduce. God’s most complex creation remains a creature of habit.
